# Tissue Flossing Around the Thigh Does Not Provide Acute Enhancement of Neuromuscular Function

**DOI:** 10.3389/fphys.2022.870498

**Published:** 2022-04-27

**Authors:** Armin H. Paravlic, Jure Segula, Kristina Drole, Vedran Hadzic, Maja Pajek, Janez Vodicar

**Affiliations:** ^1^ Faculty of Sport, University of Ljubljana, Ljubljana, Slovenia; ^2^ Science and Research Centre Koper, Institute for Kinesiology Research, Koper, Slovenia; ^3^ Faculty of Sports Studies, Masaryk University, Brno, Czechia

**Keywords:** tensiomyography (TMG), countermovement jump (CMJ), potentiation, athletic performance, ischemic preconditioning (IPC), functional performance

## Abstract

Nowadays, various methods are used for acute performance enhancement. The most recent of these is tissue flossing, which is becoming increasingly popular for both performance enhancement and rehabilitation. However, the effects of flossing on athletic performance have not been clearly demonstrated, which could be due to differences in the methodology used. In particular, the rest periods between the end of the preconditioning activity and the performance of the criterion task or assessment tools varied considerably in the published literature. Therefore, the present study aimed to investigate the effects of applying tissue flossing to the thigh on bilateral countermovement jump performance and contractile properties of vastus lateralis (VL) muscle. Nineteen recreational athletes (11 males; aged 23.1 ± 2.7 years) were randomly assigned to days of flossing application (3 sets for 2 min of flossing with 2 min rest between sets) with preset experimental pressure (EXP = 95 ± 17.4 mmHg) or control condition (CON = 18.9 ± 3.5 mmHg). The first part of the measurements was performed before and after warm-up consisting of 5 min of cycling followed by dynamic stretching and specific jumping exercises, while the second part consisted of six measurement points after flossing application (0.5, 3, 6, 9, 12, 15 min). The warm-up improved muscle response time (VL = -5%), contraction time (VL = -3.6%) muscle stiffness (VL = 17.5%), contraction velocity (VL = 23.5%), jump height (13.9%) and average power (10.5%). On the contrary, sustain time, half-relaxation time and take-off velocity stayed unaltered. Flossing, however, showed negative effects for muscle response time (F = 18.547, *p* < 0.001), contraction time (F = 14.899, *p* < 0.001), muscle stiffness (F = 8.365, *p* < 0.001), contraction velocity (F = 11.180, *p* < 0.001), jump height (F = 14.888, *p* < 0.001) and average power (F = 13.488, *p* < 0.001), whereas sustain time, half-relaxation time and take-off velocity were unaffected until the end of the study protocol regardless of condition assigned and/or time points of the assessment. It was found that the warm-up routine potentiated neuromuscular function, whereas the flossing protocol used in the current study resulted in fatigue rather than potentiation. Therefore, future studies aimed to investigate the dose-response relationship of different configurations of preconditioning activities on neuromuscular function are warranted.

## Introduction

Nowadays, several methods are used by the practitioners to acutely increase athletic performance (Wilson et al., 2013; Salvador et al., 2016). The most recent of these is tissue flossing, which is becoming increasingly popular and is used for performance enhancement or as adjunct rehabilitation therapy (Vogrin et al., 2020b; Konrad et al., 2020, 2021). In this method, tissue is compressed by wrapping a thick elastic band around a joint or muscle, resulting in partial restriction of blood flow (Konrad et al., 2021). In practice, different compression protocols are used, varying in the duration of the elastic band application, the location, the pressure applied to the covered tissue, and the method type of exercise during application (passive or active, with or without additional weight) (Vogrin et al., 2020a; Konrad et al., 2020, 2021). It is speculated that tissue flossing with a floss band involves mechanisms similar to ischemic preconditioning or blood flow restriction training (Driller et al., 2017; Konrad et al., 2021), in which reperfusion of blood to the occluded area is associated with a subsequent increase in growth hormone and catecholamine responses (Loenneke and Pujol, 2009; Loenneke et al., 2011), enhanced muscle force and contractility, and increased efficiency of excitation-contraction coupling in the working muscles (Loenneke et al., 2015). However, the effects of flossing on potentially improving athletic performance have not been clearly demonstrated (Driller and Overmayer, 2017; Vogrin et al., 2020a; Konrad et al., 2021). One explanation for the conflicting results of the published literature could be the difference in methodology used. For example, Driller and Overmayer (2017) applied the floss band around the ankle joint, whereas García-Luna Marco et al. (2020) wrapped the floss band around the knee joint, providing minimal coverage of the soft tissues. Other authors, however, applied floss band around thigh (Vogrin et al., 2020a; Konrad et al., 2020), thus covering only the soft tissue, which may have different effects on local tissue perfusion and corresponding neuromuscular function.

Neuromuscular function can be assessed by a variety of means. However, measurements of strength and power capacities such as maximal voluntary strength and power by dynamometry and vertical jump tests are most commonly used (Seitz et al., 2000; Wilson et al., 2013; Konrad et al., 2020, 2021; Haugen et al., 2021). Recent technological developments allow us to assess surrogate measures of muscle power, i.e., contractile muscle properties, using tensiomyography (TMG) without fatiguing or potentiating the measured muscle (Šimunič, 2012; Paravlić et al., 2017). TMG is based on the non-invasive and selective assessment of skeletal muscle contractile properties using a displacement sensor placed on the skin over the individual muscle belly (Valenčič, 1990). Several parameters of muscle contraction could be derived from TMG response, of which contraction time and maximal radial displacement amplitude (Dm) have been shown to be the most reliable (Šimunič, 2012; Paravlić et al., 2017) and clinically relevant (Pišot et al., 2008; Paravlić et al., 2020). In recent years, it has been used extensively to measure muscle adaptations following disuse, training processes, and various rehabilitation protocols (Pišot et al., 2008; Seijas et al., 2016; Zubac et al., 2019; Paravlić et al., 2020). Moreover, Šimunič and colleagues (Šimunic et al., 2011) demonstrated that contraction time alone, or in combination with delay time and half-relaxation time, can reliably estimate MHC-1 proportion in the vastus lateralis (VL) muscle by having standard error of estimates of 6%. Quite recently, Vogrin and colleagues (Vogrin et al., 2020a) used TMG to evaluate the effects of different degrees of pressure applied to wrap the floss band around the upper thigh on muscle contractile properties. The authors applied low and high flossing pressures as previously recommended (Loenneke et al., 2015), while performing 3 sets of 2 min of flossing with active movement followed by 2 min of rest between sets. The authors found a reduction in the contraction time of the rectus femoris muscle, followed by an improvement in the maximal voluntary isometric strength of the knee extensor muscles, suggesting neuromuscular potentiation 5 and 30 min after flossing. On the other hand, Konrad et al. (2020) found an acute increase in maximal voluntary isometric strength, but no significant effect was observed on jumping performance after a single 2-min application of flossing around thigh. Therefore, it could be hypothesized that the stimulus provided in the study by Konrad et al. (2020) was not enough to induce positive effects on vertical jump height.

Generally, effects of acute performance enhancement methods were found to depend on the rest periods between the end of the preconditioning activity (PCA) and the performance of criterion task (Wilson et al., 2013). Regardless of other modifying factors, short (3–7 min) and moderate (7–10 min) rest periods were found to be the most efficient for performance improvement following PCA (Wilson et al., 2013), so timely post measurements seems to be crucial to evaluate and optimize PCA effects. Previous studies employing flossing have conducted post measurement immediately after (Vogrin et al., 2020a) and after 5 min; after only 5 min (Konrad et al., 2020), or after 5, 15, 30 and 45 min (Driller et al., 2017). However, performing different batteries of tests at the time, including range of motion, contractile muscle properties, maximal voluntary strength and lower limb muscle power, may influence subsequent results and recognition of the optimal window for post conditioning activity performance enhancement. Studies have shown that rapid force recovery in contracting muscle after 2 min of total ischemia is dependent on oxygen availability, whereas 30-s blood reperfusion appears to be sufficient to restore muscle force production to baseline levels (Hogan et al., 1999). Hence, it would be of interest to sport practitioners to obtain more information on the acute neuromuscular functional changes after flossing as a function of time by measuring several consecutive time points from immediately after to 15 min after flossing, respectively.

Therefore, the primary purpose of the study was to investigate the acute effects of applying tissue flossing around the thigh on neuromuscular function using TMG and countermovement jump (CMJ), considering several measurement points after flossing (i.e., +0.5, +3, +6, +9, +12, +15 min). A better understanding of the effects of flossing on acute performance enhancement as well as on individual muscle contractile properties could help practitioners to develop specific pre-conditioning activities and consequently improve athletic performance on the field. We hypothesized that the use of flossing would improve the contraction time of the VL as well as the maximal jumping performance as a function of time. Therefore, we expected that the greatest effects would occur short to moderate rest periods (3–9 min) following flossing application.

## Materials and Methods

### Study Design

A randomized, crossover, counterbalanced repeated-measures designed study was conducted to investigate the effects of tissue flossing application around the thigh on neuromuscular function using TMG and CMJ. Participants were informed to refrain from alcohol and/or caffeine-based beverage consumption and to avoid strenuous workouts for at least 48 h prior to and during the study. Before the actual study begun, a familiarization trial was held to introduce all participants with the study protocol and testing procedures.

### Participants

In the conceptualization phase of the study, we conducted a power analysis using the G*Power (Faul et al., 2007). Based on previous studies with similar design we expected to find medium effects between PRE flossing and at least one POST flossing measurement point (0.55) (Vogrin et al., 2020a) with power of 0.80 and α = 0.05, two-tailed, which calculated a sample size of 16 participants. Therefore, nineteen recreationally trained university athletes (11 males; aged 23.1 ± 2.7 years, body height 174.6 ± 8.9 cm; and body mass 71.7 ± 14.2 kg) engaged in different competitive sports (team sports such as soccer, volleyball, basketball and handball, *N* = 7; and individual sports such as athletics, jiu-jitsu, gymnastics, skiing, swimming and fitness, *N* = 12) with training experience of 9.5 ± 4.3 years on average were recruited. Participants’ eligibility was determined by interview before a detailed explanation of the study protocol. Participants who had latex allergy, hypertension, venous thrombotic disease, cardio-respiratory disease, or neurological disorders, acute and or chronic neuromuscular injuries, i.e., with history of serious lower limb injuries in 12-month period prior study begun were excluded. All participants were informed about the benefits and potential risks of the study and gave written informed consent to participate in the current investigation. All procedures were carried out in accordance with the ethical standards of the 1964 Declaration of Helsinki and were approved by the Ethics Committee of (Intentionally left blank). The experimental protocol was registered on ClinicalTrials.gov, Identifier: NCT05094713.

### Procedures

**FIGURE 1:**
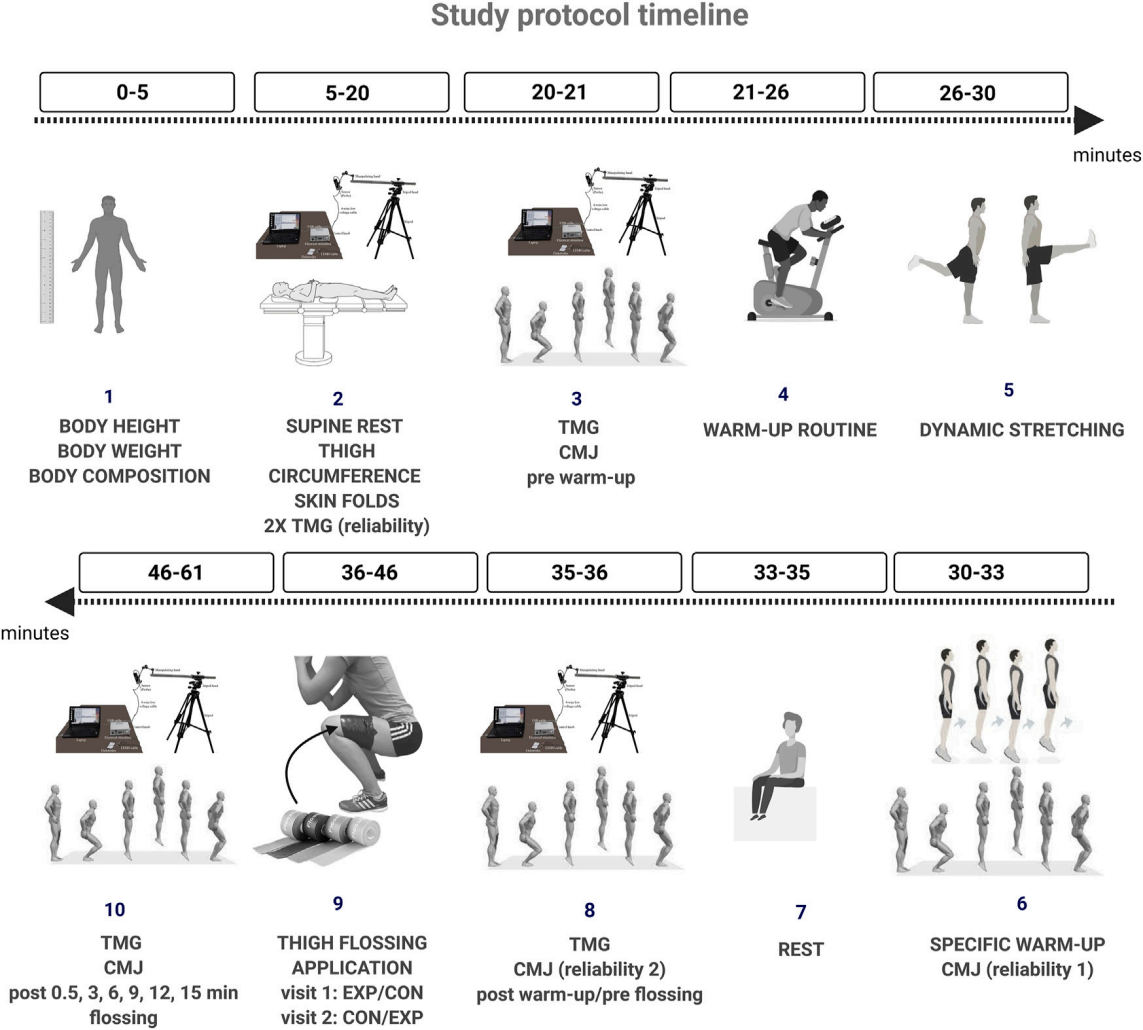
The schematic diagram of the research methodology.

Participants came to the laboratory on 2 days separated by 48 h and were randomly assigned the days for the trials with an experimental tissue flossing (EXP) using predetermined pressure or control (CON). The randomization procedure was done using the “RAND” function provided in Excel 2019 (Microsoft, Redmond, WA, United States). Study protocol is presented in Figure 1 (created with BioRender.com). Briefly, upon the arrival at the laboratory the participants’ body height and body mass were measured using a stadiometer and scale anthropometer (GPM, Model 101, Zurich, Switzerland) to the nearest 0.1 cm, while body composition was assessed with multifrequency bioelectrical impedance (InBody 720: Biospace, Tokyo, Japan). Following anthropometric assessment, participants laid down and rested for 15 min to diminish possible muscle potentiation and achieve better body fluid redistribution (Berg et al., 1993). During the resting period, upper leg length, thigh circumferences and skin folds over VL muscle of dominant leg were taken. Leg dominance was defined as the preferred leg the participants are kicking the ball with (Abazovíc et al., 2015). Immediately following a resting TMG and CMJ measurements were conducted (PRE warm-up), after which a standardized warm-up routine begun.

### Warm-Up Routine

The warm-up was divided into two parts, namely a general warm-up and a specific warm-up. The general warm-up consisted of upright cycling on stationary ergometer (Schiller, model ERG 911 plus) for 5 min at an intensity of 1 W per kg of participant body weight at a cadence of 70 revolutions per minute. A dynamic stretching protocol consisting of two sets of ten repetitions for each leg was then performed in the following order: 1) unilateral standing hip abductions and adductions at maximum amplitude; 2) unilateral standing hip flexions and extensions at maximum amplitude; and 3) dynamic hamstring stretches. The stretching tempo was controlled at 1 s for concentric and eccentric movement phases. After the general warm-up, a specific warm-up was performed consisting of three bilateral CMJ jumps with 20 s rest, followed by two sets of 10 bilateral stiff-leg ankle hops with 30 s rest between sets.

### Flossing Band Application

After the pre-test (PRE-FLOS), participants were instructed to stand still with their legs shoulder-width apart. The floss band (1.3 mm thick, 50 mm wide, 2 m long, and strength level 3), consisting of a thick elastic latex band (COMPRE Floss, Sanctband), was wrapped bilaterally around the distal third of the thigh. The floss band was applied simultaneously by two experienced kinesiologists, each wrapping one leg. The wrapping procedure began on the lateral side of the thigh, approximately 2 cm above the proximal edge of the patella. The elastic band was first pulled and then wrapped around the thigh in a circular motion, moving in a controlled manner proximally toward the hip. Each subsequent wrap overlapped the previous wrap by approximately 50% of the band (i.e., 25 mm), while the final portion of the band was secured below the last wrap. When fully applied, the floss band covered 15 cm of the thigh length on average. The applied pressure was individualized by thigh circumferences as previously recommended (Loenneke et al., 2015), whereas low target pressure was chosen for current study, showing potential to elicit the most beneficial outcomes following flossing (Vogrin et al., 2020a). The pressure was monitored by the PicoPress device (MICROLAB ELETTRONICA, Italy) on the dominant leg only. The pressure sensor was placed at the middle distance of the application of the floss band to ensure that the entire surface of the pressure sensor was always completely covered. Once the floss band was fully applied, participants were instructed to perform 10 deep squats (2s:2s for eccentric and concentric movement phase) followed by 15 leg extensions without added weight while seated on the edge of the therapeutic table (1s:1s for eccentric and concentric movement phase). After 120 s of floss band application, the band was unwrapped and participants had 120 s of rest during which they were instructed to stand up and take at least 30 steps in the lab. During the last 30 s of rest, the floss band was applied again as reported elsewhere (Vogrin et al., 2020a). This procedure was repeated three times while participants rated their rate of perceived exertion induced by ischemia, before and after each set using 10-points Borg scale.

### Tensiomyography Assessment

The contractile properties of the VL muscle were assessed using the non-invasive TMG method. For this purpose, the VL of the dominant side was measured while supine at rest at a knee angle set to 30° of knee flexion. The knee angle and the resting state of the leg were secured by knee foam pads provided by the TMG manufacturer (TMG-BMC, Ljubljana, Slovenia). The well-established methodology was used as previously described (Šimunič, 2012; Paravlić et al., 2020). Briefly, following an electrically induced isometric twitch, the radial displacement of the muscle belly was recorded at the skin surface using a sensitive digital displacement sensor (TMG-BMC, Ljubljana, Slovenia). The sensor was set perpendicular to the skin normal plane above the muscle belly as recommended (Delagi et al., 2011). The rounded (5-cm diameter) self-adhesive cathode and anode (Axelgaard, Aarhus, Denmark) were set 5 cm distally and 5 cm proximally to the measuring point, on all muscles assessed. Electrical stimulation was applied through a TMG-100 System electro stimulator (TMG-BMC d.o.o., Ljubljana, Slovenia) with a pulse width of 1 ms and an initial amplitude of 20 mA. During each measurement, the amplitude was progressively increased by 20 mA increments until there was no further increase in the amplitude of the TMG response, which was usually accompanied by the maximal stimuli of 110 mA. Rest periods between two stimuli of 30 s were given between each stimulus to minimize the effects of fatigue and potentiation. More detailed testing procedures were previously described elsewhere (Šimunič, 2012; Paravlić et al., 2017). From two maximal twitch responses, several TMG parameters were calculated, as follows: delay time as time from an electrical impulse to 10% of the maximal displacement amplitude (Dm); contraction time as time from 10 to 90% of Dm; sustain time as time from 50 to 50% of Dm; and half-relaxation time as time from 90 to 50% of Dm. Additionally, the index representing the velocity of contraction (Vc) (Pereira et al., 2018) and MHC-I proportion (in %) (Loturco et al., 2016) were calculated.

### Jumping Performance Assessment

Jumping performance was assessed by CMJ test, using bilateral force plate (model 9260AA6, Kistler, Winthertur, Switzerland). Before the actual test a 2-3 warm-up bilateral squat jumps and bilateral CMJ with hands placed on the hips were executed, followed by actual maximal jump trials. Test execution was supervised from an experienced researcher to improve proficiency in jumping technique. Jumps were repeated after a 30-s rest period, until three valid CMJ jumps were achieved. Participants were instructed and verbally encouraged to maximize their jump height. Finally, the jump with the maximal achieved height calculated from take of velocity was taken for further analysis (Moir, 2008), as an primary CMJ outcome. Additionally, a vertical take-off velocity and average power were reported.

### Statistical Analysis

All data are presented as mean ± SD. All statistical analysis was done with SPSS statistical software (version 27.0, IBM Inc., Chicago, United States). Descriptive statistics were used to summarize demographic characteristics of participants and outcomes. Normality was confirmed by visual inspection and using the Shapiro-Wilk test, while the homogeneity of variances was tested using Levene’s test for all dependent variables. To investigate the effects of warm-up on TMG and CMJ performance parameters, the student t-test for paired samples was used for the experimental visit only. Main effects of tissue flossing procedure on TMG and CMJ performance were studied with a repeated-measures General Linear Model with time (PRE and POST 0.5, POST +3, POST +6, POST +9, POST +12, POST +15) as within factor, while condition (EXP vs. CON) was used as between factor. In the case of significant effects were found for muscle, time effects, or 2-way interactions (three-way interactions were excluded from the analysis), post hoc comparisons with Bonferroni corrections were applied to address significant PRE-to-POST differences for each variable independently. Sphericity (homogeneity of covariance) was verified by the Mauchly’s test. When the assumption of sphericity was not met, the significance of the F-ratios was adjusted according to the Greenhouse-Geisser procedure. The magnitude of effect for all dependent variables was reported as partial eta squared (η2). Additionally, *Cohens’ d* (ES) was used to assess magnitude of change from PRE flossing to each POST period of assessment and was interpreted as: *trivial*: <0.20, *small*: 0.20–0.50, *moderate*: 0.50–0.80, or *large*: >0.80 (Cohen, 1988).

To determine reliability of the data presented, intra-trial measurements (separated by 3 min) during familiarization visit were evaluated. The relative reliability of all dependent variables was estimated using the intra-class correlation coefficient (ICC), two-way random model (consistency type). ICC values were considered as very high if > 0.90, high if between 0.70 and 0.89, and moderate if between 0.50 and 0.69 (Atkinson and Nevill, 1998; Paravlić et al., 2017). In addition, the standard error of estimate (SEM) followed by the coefficient of variation (CV) were calculated as measures of absolute reliability, which indicates within-subject variation, as previously suggested in the literature (Hopkins, 2000). To further address the reliability issues of the data, bias and random error were calculated, followed by the minimal detectable change (MDC) calculation, a measure of the minimal amount of change unrelated to variations of the assessment that can be considered as clinically relevant. Statistical significance for all analysis conducted was accepted at *p* ≤ 0.05.

## Results

### Demographic Characteristic of Participants

A total of 19 participants were measured in a randomised cross-over trial. They had a body fat percentage of 13.8 ± 6.8% and a skeletal muscle mass of 35.6 ± 8.9 kg. In addition, thigh circumference averaged 50.5 ± 4.7 cm, while skin fold over the VL TMG measurement points was 9.6 ± 3.9 mm.

### The Reliability Analysis

The reliability analysis data were presented in Table 1. The reliability analysis showed a high to very high average ICC (relative reliability score) for all dependent variables, ranging from 0.85 to 0.99 for VL Vc and CMJ height, respectively. Measures of absolute variability, presented as CV% ranged from 1.1% (take-off velocity) to 10.1% (VL relaxation time).

**TABLE 1:** Between Time 1 and Time 2 reliability analysis of the TMG and CMJ performance parameters.

Variable		Time 1	Time 2	P_ANOVA_	CV (%)	MDC	SEM	ICC (95%CI)	Cronbach’s alpha
Vastus Lateralis	Delay time (ms)	20.25 ± 1.19	20.38 ± 1.02	0.171	1.1	0.56	0.20	0.966 0.911 0.987	0.966
	Contraction time (ms)	20.37 ± 1.85	20.24 ± 1.73	0.253	1.5	0.69	0.25	0.98 0.949 0.992	0.980
	Sustain time (ms)	43.06 ± 21.19	38.91 ± 13.17	0.188	9.2	19.56	7.08	0.837 0.576 0.937	0.837
	Relaxation time (ms)	16.77 ± 12.09	16.67 ± 11.38	0.912	10.1	5.81	2.10	0.967 0.914 0.987	0.967
	Maximal displacement amplitude (mm)	5.21 ± 1.27	5.23 ± 1.07	0.899	6.9	0.39	1.08	0.886 0.704 0.956	0.886
	Contraction velocity (mm/ms)	0.13 ± 0.03	0.13 ± 0.03	0.905	1.3	0.03	0.01	0.854 0.621 0.944	0.854
CMJ parameters	CMJ height (cm)	37.01 ± 7.49	37.07 ± 7.65	0.837	2.0	1.85	0.67	0.992 (0.980–0.997)	0.992
	Take-off velocity (m/s)	2.68 ± 0.26	2.68 ± 0.27	0.862	1.0	0.17	0.06	0.991 0.978 0.997	0.991
	Average power (W)	1960.16 ± 605.74	1978.64 ± 601.66	0.486	2.6	156.14	56.50	0.991 (0.977–0.997)	0.991

P_ANOVA_—P-value of repeated measures analysis of variance; CV—within-subject coefficient of variation; MDC—minimal detectable change; SEM—standard error of estimate; ICC (95% CI)—intra-class correlation coefficient with 95% confidence intervals; 19 subjects in total were assessed at Time 1 and Time 2

### Effects of Warm-Up on TMG and CMJ Performance

Except for VL sustain time, VL relaxation time, and take-off velocity, there was a significant positive effect of warm-up for all variables assessed as follows: VL delay time (*p* < 0.001, *large* ES = - 1.08), VL contraction time (*p* = 0.001, *moderate* ES = - 0.76), VL Dm (*p* < 0.001, *large* ES = 1.00), VL Vc (*p* < 0.001, *large* ES = 1.18), CMJ height (*p* < 0.001, *large* ES = 1.91) and average power (*p* < 0.001, *large* ES = 1.43) (Table 2).

**TABLE 2:** Comparison of vastus lateralis muscle contractile properties and jumping performance measures from PRE to POST warm-up protocol.

Variable	PRE-warm-up	POST-warm-up	*t* value	*p* value	ES	CI-ll	CI-ul
	Mean ± SD	Mean ± SD					
VASTUS LATERALIS							
Delay time (ms)	20.15 ± 1.38	20.09 ± 0.99	4.708	**<0.001**	1.08	0.501	1.641
Contraction time (ms)	20.19 ± 2.13	19.46 ± 1.66	3.294	**0.004**	0.76	0.236	1.260
Sustain time (ms)	42.48 ± 27.44	36.75 ± 15.31	1.136	0.271	0.26	−0.200	0.715
Half-relaxation time (ms)	20.10 ± 25.91	15.75 ± 13.93	0.890	0.385	0.20	−0.253	0.656
Maximal displacement amplitude (mm)	4.95 ± 0.98	5.82 ± 1.45	−4.341	**<0.001**	−1.00	−1.540	−0.433
Contraction velocity (mm/ms)	0.12 ± 0.02	0.15 ± 0.04	−5.129	**<0.001**	−1.18	−1.757	−0.577
CMJ PARAMETERS							
CMJ height (cm)	33.48 ± 6.73	38.12 ± 8.09	−8.309	**<0.001**	−1.91	−2.661	−1.133
Take-off velocity (m/s)	2.55 ± 0.25	2.97 ± 1.08	−1.676	0.111	−0.38	−0.846	0.087
Average power (W)	1845.99 ± 537.59	2042.37 ± 608.46	−6.224	**<0.001**	−1.43	−2.064	−0.773

CMJ—countermovement jump; NA—not applicable; CI-ll—lower limit of confidence interval; CI-ul—upper limit of confidence interval; Bolded values—significant difference

### Comparison of Applied Pressure and Subjective Ratings of Perceived Exertion During Flossing Application Procedure

Applied pressure during flossing application significantly differ between conditions (*p* < 0.001, large ES = 5.9; EXP = 95 ± 17.4 mmHg vs. CON = 18.9 ± 3.5 mmHg).

There was a main effect of time (*p* < 0.001; η2 = 0.860) and time*condition interaction (*p* < 0.001; η2 = 0.589) on participants’ subjective ratings of pain during thigh flossing. Post-hoc analysis showed that compared to the control condition, the participants during the experimental condition rated their perceived pain significantly higher (EXP vs. CON; 5.6 ± 1.5 vs. 1.9 ± 1.2 on average) after each set: after the first set (*p* < 0.001, large ES = 2.84), the second set (*p* < 0.001, large ES = 2.78), and the third set (*p* < 0.001, large ES = 2.23).

### Effects of Tissue Flossing on TMG and CMJ Performance

There was a main effect of time for all assessed TMG variables as follows: delay time (*p* < 0.001; η2 = 0.340), contraction time (*p* < 0.001; η2 = 0.293), Dm (*p* < 0.001; η2 = 0.189) and Vc (*p* < 0.001; η2 = 0.237). In contrary, time effect for relaxation time and sustain time was not significant. There was no significant time*condition interaction for any variable assessed (Supplementary Table S1).

Post-hoc analysis showed that TMG parameters were significantly altered at POST measurement points compared to PRE-flossing regardless of condition (Table 3). Thus, compared to PRE, delay time showed decrease at POST+0.5 min only (EXP: PC = 1.8%, *p* = 0.002; CON: PC = 2.2, *p* = 0.002); contraction time showed increase at POST+6 min (EXP: PC = 2.1%, *p* = 0.002; CON: PC = 1.6, *p* = 0.002); POST+9 min (EXP: PC = 3.8%, *p* < 0.001; CON: PC = 2.7, *p* < 0.001); POST+12 min (EXP: PC = 4.7%, *p* < 0.001; CON: PC = 3.0, *p* < 0.001); and POST+15 min (EXP: PC = 4.0%, *p* < 0.001; CON: PC = 3.1, *p* < 0.001), respectively. Furthermore, the Dm significantly decreased at POST+0.5 (EXP: PC = -7.8%, *p* < 0.001; CON: PC = -8.6, *p* < 0.001); and POST+3 min (EXP: PC = -13.1%, *p* = 0.001; CON: PC = -6.2, *p* = 0.004), and same occurred for Vc at POST+0.5 (EXP: PC = -7.9%, *p* = 0.002; CON: PC = -7.7, *p* = 0.002); and POST+3 min (EXP: PC = -14.3%, *p* = 0.001; CON: PC = -6.3, *p* = 0.004), respectively (Table 3).

**TABLE 3:** Comparison of all post measures (0.5, 3, 6, 9, 12 and 15-min) to pre-flossing intervention values. Data are presented as mean ± SD.

Parameters	PRE		POST+ 0.5		POST +3		POST +6		POST +9		POST +12		POST +15	
	FLOS	Control	FLOS	Control	FLOS	Control	FLOS	Control	FLOS	Control	FLOS	Control	FLOS	Control
	Mean ± SD	Mean ± SD	Mean ± SD	Mean ± SD	Mean ± SD	Mean ± SD	Mean ± SD	Mean ± SD	Mean ± SD	Mean ± SD	Mean ± SD	Mean ± SD	Mean ± SD	Mean ± SD
VASTUS LATERALIS														
Delay time (ms)	20.1 ± 1.0	20.3 ± 1.0	19.7 ± 1.0^*^	19.9 ± 1.1^*^	20 ± 1.1	19.9 ± 0.9	20.1 ± 1.1	20.1 ± 1.1	20.2 ± 1.2	20.4 ± 1.2	20.2 ± 1.2	20.4 ± 1.0	20.4 ± 1.0	20.6 ± 1.3
Contraction time (ms)	19.5 ± 1.7	19.8 ± 1.7	19.3 ± 1.8	19.6 ± 1.8	20 ± 1.8	19.9 ± 2.1	19.9 ± 1.8^*^	20.1 ± 1.9*	20.2 ± 1.8*	20.3 ± 1.9*	20.4 ± 1.8*	20.4 ± 1.9*	20.2 ± 1.7*	20.4 ± 2.1*
Sustain time (ms)	36.8 ± 15.3	37.1 ± 14.5	34.2 ± 10.4	37.6 ± 19.4	34.7 ± 8.6	43.1 ± 32.0	35.9 ± 11.2	35.1 ± 8.3	38.3 ± 15.6	33.7 ± 5.0	43.1 ± 21.2	39 ± 20.1	38.9 ± 13.2	40.3 ± 15.4
Half-relaxation time (ms)	15.7 ± 13.9	15.5 ± 12.6	13.1 ± 9.2	16.4 ± 17.9	12.9 ± 7.6	21.1 ± 30.1	14.3 ± 10.1	13 ± 6.3	16.3 ± 14.1	11.6 ± 3.1	20.7 ± 19.9	16.6 ± 18.2	16.7 ± 11.4	17.7 ± 13.0
Maximal displacement amplitude (mm)	5.8 ± 1.4	6 ± 2.1	5.4 ± 1.5^*^	5.5 ± 2.1^*^	5.1 ± 1.4^*^	5.6 ± 2.6^*^	5.1 ± 1.2	5.6 ± 2.8	5.2 ± 1.3	5.8 ± 2.9	5.2 ± 1.3	5.7 ± 2.9	5.2 ± 1.1	5.7 ± 2.6
Contraction velocity (mm/ms)	0.15 ± 0.04	0.15 ± 0.05	0.14 ± 0.04^*^	0.14 ± 0.06^*^	0.13 ± 0.03^*^	0.14 ± 0.07^*^	0.13 ± 0.03	0.14 ± 0.07	0.13 ± 0.03	0.14 ± 0.07	0.13 ± 0.03	0.14 ± 0.07	0.13 ± 0.03	0.14 ± 0.07
CMJ PARAMETERS														
CMJ height (cm)	38.1 ± 8.1	38.2 ± 8.6	36.3 ± 7.9^*^	36.7 ± 8.0^*^	36.3 ± 8.2^*^	36.9 ± 8.4^*^	36.8 ± 8.1^*^	36.5 ± 8.0*	36.2 ± 8.0*	36.7 ± 8.2*	36.1 ± 8.1*	36 ± 7.4*	36.4 ± 7.5*	36 ± 8.0*
Take-off velocity (m/s)	2.97 ± 1.08	2.72 ± 0.03	2.66 ± 0.28	2.67 ± 0.28	2.65 ± 0.29	2.68 ± 0.30	2.67 ± 0.29	2.66 ± 0.29	2.65 ± 0.29	2.67 ± 0.29	2.65 ± 0.29	2.65 ± 0.27	2.66 ± 0.27	2.64 ± 0.29
Average power (W)	2042.4 ± 608.5	2032.2 ± 598.2	1939.6 ± 607.3^*^	1944.4 ± 595.5^*^	1945.8 ± 601.8^*^	1973.7 ± 576.8^*^	1971 ± 605.1^*^	1968.6 ± 597.7*	1925.4 ± 576.6*	1939.5 ± 549.3*	1914 ± 588.6*	1922.6 ± 549.6*	1971.8 ± 575.1*	1919.9 ± 572.9*

CMJ—countermovement jump; FLOS—experimental group; significantly different compared to PRE (*)

Considering CMJ performance parameters, a main effect of time was found for CMJ height (*p* < 0.001; η2 = 0.293) and average power (*p* < 0.001; η2 = 0.273), while take-off velocity was not affected by flossing application. Moreover, time*condition interactions were not identified (Supplementary Table S1).

Post-hoc analysis showed that CMJ height and average power were negatively altered from PRE-FLOS to all POST time points (all *p* < 0.001), while take-off velocity stayed unchanged, regardless of which condition participants were assigned to (Table 3).

## Discussion

The present study was primarily conducted to investigate the effects of applying tissue flossing to the thigh on neuromuscular function using TMG and CMJ. The results of the present study show that: 1) the warm-up had a positive effect on neuromuscular function, as evidenced mainly by reduction in delay time and contraction time of the VL muscle, while Dm, contraction velocity and jump height increased, and 2) the preconditioning activity of applying tissue flossing around the thigh showed no difference in the neuromuscular function parameters compared to the control condition.

### Effects of Warm-Up Routine on Neuromuscular Function

There was a significant positive effect of warm-up routine on the improvement of neuromuscular function, consistent with the previous literature (Bishop, 2003b; McGowan et al., 2015; Redd et al., 2021). Given the high reliability of the data obtained (Table 1), our results support the usefulness of both CMJ and TMG as tools for monitoring acute changes in neuromuscular function. Primarily, the reduction in delay time (VL–5%) and contraction time (VL–3.6%) were observed, while muscle stiffness (VL–17.5%), contraction velocity (VL–23.5%), jump height (13.9%), take-off velocity (16.4%), and average maximal power (8.8%) each increased. Our results considering TMG measurements are supported by recent findings of Abazovic and colleagues (Abazovic et al., 2022). Latter authors investigated the effect of the post-activation potentiation protocol (i.e., 5 × 5 s of maximal voluntary isometric contraction) on the contractile parameters of the VL and vastus medialis muscles using TMG. The authors found a potentiation during the first 2 min after the activation stimulus, as evidenced by a shortened contraction time in both muscles. Thus, authors confirmed that TMG is sensitive method to detect post-activation potentiation effects with a sensor mounted directly above the muscle belly. Moreover, our results are consistent with a recently published study (Redd et al., 2021) that examined the TMG response to various warm-up protocols, including dynamic, plyometric, and small-sided games warm-up. Although measuring other muscles (e.g., rectus femoris and biceps femoris), the authors found similar changes from pre to post the warm-up protocols, as evidenced by reduction in contraction time (RF–5%; BF–6.6%), and delay time (RF–5.6%; BF–5.1%) (Redd et al., 2021). The changes in muscle contraction properties and other strength-related performance measures such as sprinting and jumping activities can be attributed to various central and peripheral mechanisms (Hamada et al., 2000; Bishop, 2003b; McGowan et al., 2015; Seitz and Haff, 2016).

The mechanisms underlying effectiveness of active warm-up on the neuromuscular function are mainly attributed to temperature-related factors, such as increased blood flow to working musculature leading to increased oxygen delivery, acceleration of rate-limiting oxidative reactions, increased anaerobic metabolism, and increased nerve conduction velocity, while viscous tissue resistance decreases, leading to decreased intrafascial resistance and consequent performance improvement (Bishop, 2003a; Behm et al., 2021). Evidence suggests that improved muscle performance following warm-up and/or PCA may be explained by the post-activation potentiation phenomenon governed by greater cortical output and subsequent recruitment of higher order motor units (Hamada et al., 2000; Tillin and Bishop, 2009; Wilson et al., 2013; Seitz and Haff, 2016; Wallace et al., 2019). In addition, numerous metabolic changes take place in a working musculature, such as adenosine triphosphate turnover, muscle bridge cycling rate, and elevated oxygen uptake kinetics, which enhance neuromuscular function (Hamada et al., 2000; McGowan et al., 2015).

### Effects of Tissue Flossing on Neuromuscular Function

Compared with the known athletic performance improvements after various warm-up routines (Bishop, 2003b; McGowan et al., 2015), the effects of flossing are equivocal (Driller and Overmayer, 2017; Konrad et al., 2020, 2021). To the best of authors knowledge, four studies have investigated the effects of flossing on lower limb muscle power by using CMJ assessment (Driller et al., 2017; García-Luna Marco et al., 2020; Konrad et al., 2020; Mills et al., 2020), while only two studies have measured contractile muscle properties with TMG (Vogrin et al., 2020a; 2020b). We found significant negative alterations in all TMG parameters assessed, that were coupled with significantly reduced CMJ performance immediately after flossing (−4.3%) and maintained this trend until the end of the study protocol (−5.1%), suggesting that muscle fatigue rather than potentiation occurred. These results differ from previous findings (Driller et al., 2017; García-Luna Marco et al., 2020; Mills et al., 2020) that reported no changes in CMJ peak force (Driller et al., 2017) or CMJ height (Konrad et al., 2020) or found a significant increase in CMJ height for 17.4% (Driller and Overmayer, 2017) and 11.1% (García-Luna Marco et al., 2020) on average. One explanation for the conflicting results compared with previous studies may be the difference in methodology (i.e., different flossing protocols used and participant characteristics). Driller and Overmayer (Driller and Overmayer, 2017) applied floss band around the ankle joint, whereas García-Luna Marco et al. (2020) wrapped the floss band around the knee joint, thus, minimally covering soft tissue, rather than just the soft tissue as we did.

The underlying mechanism of tissue flossing application have been related to those observed after blood flow restriction interventions (Driller et al., 2017; Konrad et al., 2021), including increased anaerobic muscle metabolism (Loenneke and Pujol, 2009), greater hormonal secretion (Loenneke et al., 2011), and spinal excitability (Loenneke et al., 2015). However, the effects of ischemic preconditioning have been shown to be equivocal in terms of athletic performance enhancement (Salvador et al., 2016). Similar to our findings, there are several studies demonstrating a fatiguing effect of ischemic preconditioning on subsequent performance (Howell et al., 2001; Salvador et al., 2016; Husmann et al., 2018). For example, Howell and colleagues (Howell et al., 2001), found a 20% reduction of vertical jump performance after 90 s of occlusion of the femoral artery. Alike, Husmann et al. (2018) reported a decline in maximal voluntary torque and contractile properties of the quadriceps muscle measured by twitch torque in response to paired electrical stimuli, suggesting the onset of central and peripheral muscle fatigue following ischemic preconditioning (Karabulut et al., 2010). The authors suggested that ischemic treatment exacerbates exercise-induced accumulation of fatigue-related metabolites, preventing recovery of the muscle contractile properties during rest periods between sets, and providing a possible explanation for onset of the peripheral muscle fatigue (Husmann et al., 2018). Although our warm-up protocol fulfilled its main purpose and increased neuromuscular performance, it appears that an additional 3 sets of tight flossing resulted in muscle fatigue. Therefore, further studies are warranted to investigate the dose-response relationship of different configurations of warm-up and flossing protocols on neuromuscular function, considering their volume and intensity.

A review of the previous literature on the effects of tissue flossing on neuromuscular function, revealed differences in the population assessed in terms of sex, training experience, fitness level, and health status, which could lead to conflicting results (Rixon et al., 2007; Wilson et al., 2013). For example, Konrad et al. (2020) and García-Luna Marco et al. (2020) recruited only male participants, whereas Driller and Overmayer (2017) including us, recruited females as well. Several studies (Rixon et al., 2007; Tsolakis et al., 2011; Wilson et al., 2013) have found sex to be a modifying factor in the beneficial effects of the post-activation potentiation routine on later performance measures of strength and power, consistently reporting less favourable effects for female than for male participants (Rixon et al., 2007; Tsolakis et al., 2011; Wilson et al., 2013). The observed differences in the effects of preconditioning activity could be due to greater fat-free mass and a higher proportion of fast-twitch muscle fibres (i.e., type II) in male compared with females (Staron et al., 2000; CHIU et al., 2003). CHIU et al. (2003) suggested that individuals with a higher proportion of type II muscle fibers may respond faster to preconditioning activity because type II fibres shorten faster compared with slow-twitch muscle fibers, due to their higher myosin ATPase activity (Bárány, 1967) and thus can generate more force (Bárány, 1967). A direct comparison between high and low jumpers in our sample showed that high jumpers were mostly male, heavier, had less body fat and significantly more muscle mass, while they produced more average power than low jumpers (Supplementary Table S2). Moreover, the estimation of MHC-I content used in current study showed no difference between these two groups, which prevents us from confirming findings considering the effects of MHC content on jumping performance improvements after post-activation potentiation (Hamada et al., 2000; CHIU et al., 2003). It is possible that the equation proposed by Šimunic et al. (2011) to calculate MHC-I content was not sensitive enough to distinguish between high and low jumpers, considering the variability of MHC-I content estimated in our sample (Supplementary Table S2).

Finally, it is important to point out the limitations of the present study. First, we had no knowledge of the actual blood flow in the active muscles during the flossing, although the pressure was individually adjusted when the floss band was applied. However, we measured the subjective response to perceived exertion, which has been shown to be a valid indicator of the degree of blood flow restriction (Loenneke et al., 2014). Second, we did not monitor metabolic responses in the VL muscle, whereas the dose-response of ischemic preconditioning on subsequent performance depends on oxygen availability (Hogan et al., 1999). Therefore, future studies that aim to investigate the effects of flossing on subsequent performance should employ comprehensive technology to monitor physiological responses within the working musculature. Furthermore, a dose-response relationship of floss band application is warranted considering different pressure levels, preconditioning activity (with or without added weight and/or their combination), treatment duration, and sets configuration.

## Conclusion

The warm-up routine was found to potentiate neuromuscular function, whereas the flossing protocol used in the current study resulted in fatigue rather than potentiation. Therefore, future studies aimed to investigate the dose-response relationship of different configurations of preconditioning activities (with or without added weight and/or their combination; treatment duration, number of sets) on neuromuscular function are warranted.

## Data Availability

The raw data supporting the conclusion of this article will be made available by the authors, without undue reservation.
