# Supplementary material for: Tissue Flossing Around the Thigh Does Not Provide Acute Enhancement of Neuromuscular Function
Source: Front Physiol. 2022 Apr 27;13:870498. doi: 10.3389/fphys.2022.870498 (PMC9091176; doi:10.3389/fphys.2022.870498)
Supplement: Supplementary file 1 [file Table2.docx]

**Table 1.** Main effects of TMG and CMJ parameters studied with General linear model for repeated measures for time (PRE-flossing, POST 0.5, POST + 3, POST + 6, POST + 9, POST + 12, POST + 15) as within factor and group (EXP vs. CON) as between factor.

| TMG  parameters |  | **Interactions** |
| --- | --- | --- |
|  | Time | Time*Group |
|  | F value; p value; [η^2^] | F value; p value; [η^2^] |
| Delay time (ms) | **18.547; <0.001; [0.340]** | 1.116; 0.354; [0.030] |
| Contraction time (ms) | **14.899; <0.001; [0.293]** | 0.872; 0.459; [0.024] |
| Sustain time (ms) | 1.694; 0.161; [0.045] | 0.321; 0.848; [0.009] |
| Relaxation time (ms) | 1.931; 0.096; [0.051] | 0.815; 0.534; [0.022] |
| Maximal displacement amplitude (mm) | **8.365; <0.001; [0.189]** | 1.018; 0.398; [0.027] |
| Contraction velocity (mm/ms) | **11.180; <0.001; [0.237]** | 0.933; 0.438; [0.025] |
| CMJ height (cm) | **14.888; < 0.001; [0.293]** | 1.273; 0.283; [0.034] |
| Take-off velocity (m/s) | 2.192; 0.147; [0.057] | 1.027; 0.320; [0.028] |
| Average power (W) | **13.488; <0.001; [0.273]** | 1.440; 0.223; [0.038] |
| TMG – Tensiomyography; Td – time delay; Tc – contraction time; Ts – sustain time; Dm – maximal radial displacement amplitude; **Bolded value** – significant effect (p < 0.05); | | |

## Comparison between low and high jumpers’ groups

The comparison of low and high jumpers’ group was investigated by the student t-test for independent samples. The cut-of value was based on the median CMJ height (i.e., low jumpers: ≤ 35.9 cm; high jumpers: > 35.9).

Results showed that high jumpers’ group had significantly higher body mass (p = 0.042, large ES = -0.96), body height (p = 0.008, large ES = -1.31) and skeletal muscle mass (p = 0.001, large ES = -1.73), while having lower values of total body fat (p < 0.001, large ES = 2.4). However, groups did not differ in TMG derived parameters (Table 2)

**Table 2.** Comparison of socio-demographic, antrhopometric and performance related parameters between low and high jumpers group

| **Variable** | **Low Jumpers** | **High Jumpers** |  |  |  |  |  |
| --- | --- | --- | --- | --- | --- | --- | --- |
|  | Mean ± SD | Mean ± SD | *t* value | *p* value | ES | CI-ll | CI-ul |
| **SOCIO-DEMOGRAPHIC CHARACTERISTICS** |  |  |  |  |  |  |  |
| Age | 22 ± 1.6 | 24.2 ± 3.2 | -1.96 | 0.067 | NA |  |  |
| Training experience | 8.8 ± 3.1 | 10.3 ± 5.4 | -0.77 | 0.452 | NA |  |  |
| Sex (female/male) | 2/8 | 0/9 |  |  |  |  |  |
| **BODY COMPOSITION** |  |  |  |  |  |  |  |
| Body mass (kg) | 65.5 ± 13.6 | 78.5 ± 12 | -2.199 | **0.042** | -1.01 | -1.96 | -0.04 |
| Body height (cm) | 169.7 ± 8.1 | 179.9 ± 6.6 | -2.986 | **0.008** | -1.37 | -2.37 | -0.35 |
| Body mass index | 22.6 ± 3.1 | 24.2 ± 2.8 | -1.155 | 0.264 |  |  |  |
| Total body fat (%) | 18.8 ± 4.9 | 8.3 ± 3.3 | 5.458 | **<0.001** | 2.51 | 1.26 | 3.72 |
| Skeletal muscle mass (kg) | 29.9 ± 6.4 | 41.9 ± 6.9 | -3.932 | **0.001** | -1.81 | -2.87 | -0.70 |
| Thigh circumference (cm) | 49.3 ± 4.7 | 51.9 ± 4.5 | -1.247 | 0.229 | NA |  |  |
| **VASTUS LATERALIS** |  |  |  |  |  |  |  |
| Delay time (ms) | 21 ± 1.3 | 21.3 ± 1.6 | -0.362 | 0.722 | NA |  |  |
| Contraction time (ms) | 19.8 ± 2.8 | 20.6 ± 0.8 | -0.83 | 0.418 | NA |  |  |
| Sustain time (ms) | 49.5 ± 37.2 | 34.7 ± 3.5 | 1.182 | 0.254 | NA |  |  |
| Half-relaxation time (ms) | 27 ± 35 | 12.5 ± 2.6 | 1.237 | 0.233 | NA |  |  |
| Maximal displacement amplitude (mm) | 5.2 ± 1.0 | 4.7 ± 0.9 | 0.959 | 0.351 | NA |  |  |
| Contraction velocity (mm/ms) | 0.13 ± 0.02 | 0.11 ± 0.02 | 1.430 | 0.171 | NA |  |  |
| MHC I (%) | 31.8 ± 49.3 | 16.5 ± 7.2 | 0.921 | 0.37 | NA |  |  |
| **CMJ PARAMETERS** |  |  |  |  |  |  |  |
| CMJ height (cm) | 32.5 ± 2.6 | 44.4 ± 7.4 | -5.13 | **<0.001** | -2.36 | -3.53 | -1.14 |
| Take-off velocity (m/s) | 3.0 ± 1.5 | 2.9 ± 0.2 | -5.325 | **<0.001** | -2.45 | -3.64 | -1.21 |
| Average power (W) | 1642 ± 428.7 | 2487.2 ± 450.8 | -3.81 | **0.001** | -1.75 | -2.81 | -0.66 |
| CMJ – countermovement jump; NA – not applicable; CI-ll – lower limit of confidence interval; CI-ul – upper limit of confidence interval; | | | | | | | |
